# Supplementary material for: Proposing an Optimal Occlusal Angle for Minimizing Masticatory and Cervical Muscle Activity in the Supine Position: A Resting EMG and Mixed-Effects Modeling Study
Source: Medicina (Kaunas). 2025 Jul 15;61(7):1274. doi: 10.3390/medicina61071274 (PMC12300043; doi:10.3390/medicina61071274)
Supplement: Supplementary file 1 [file medicina-61-01274-s001.zip › medicina-3737573-supplementary.pdf]

medicina

MDPI

Supplementary Materials:

Figure S1. Flow diagram

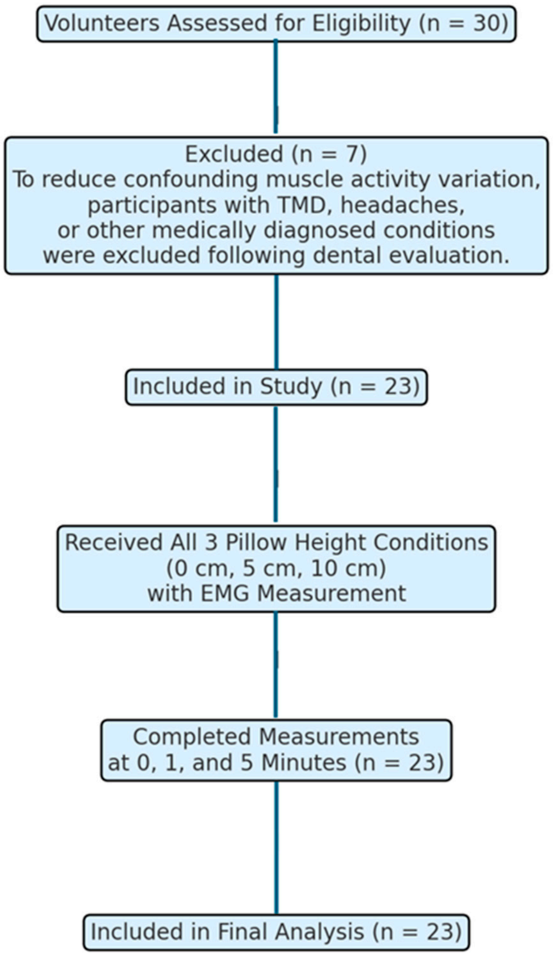

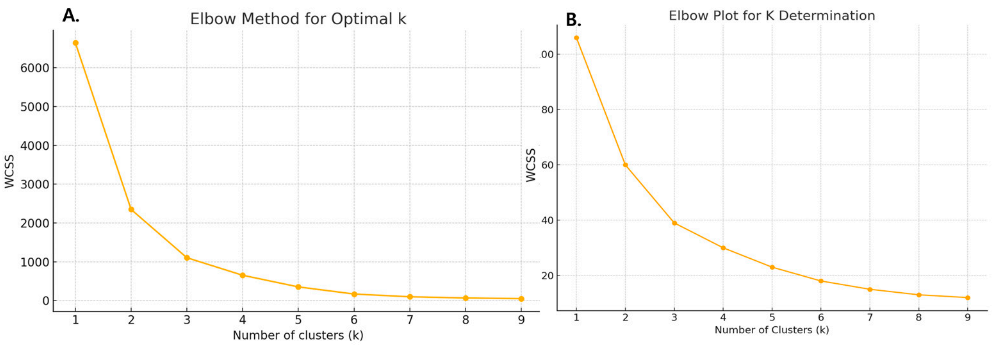

Figure S2. Elbow plot for determining the optimal number of clusters (k).

(A) Elbow plot illustrating the within-cluster sum of squares (WCSS) across k values ranging from 1 to 9. The clustering analysis was based on occlusal angle, masseter, and temporalis resting muscle ratios. (B) A clear inflection point is observed at k = 2, suggesting that two clusters optimally capture

the natural structure of the data. This supports the presence of two distinct groups, potentially corresponding to low and high muscle activation patterns in the supine position.

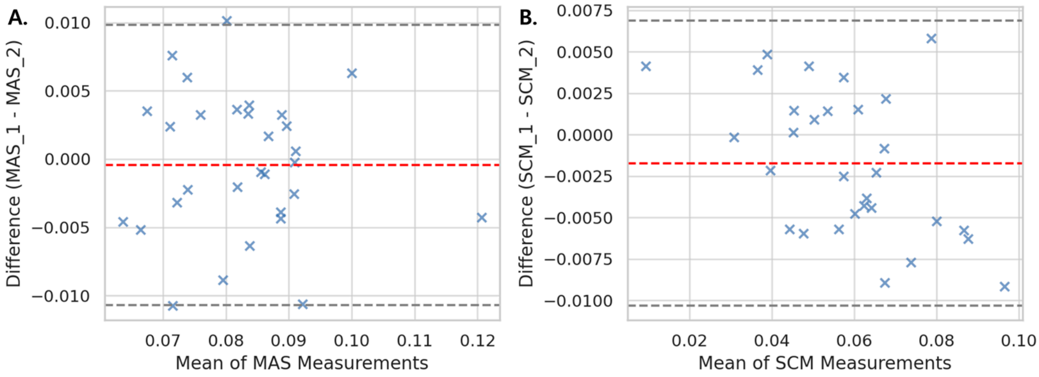

Figure S3. Bland–Altman plots for repeated electromyography measurements of masseter (MAS) and sternocleidomastoid (SCM) muscles.

(A) MAS shows small mean differences and narrow limits of agreement between two repeated measurements, indicating high measurement reliability. (B) SCM demonstrates similar reproducibility, with most data points falling within ±1.96 standard deviations of the mean difference.

Table S1. Summary of Linear Mixed-Effects Model (LMM) Results for Each Muscle (MAS, TEM, SCM, and PVM)

Muscle	Fixed Effect	β Coefficient	Standard Error	t-value	p-value
MAS	OA	0.0034	0.0012	2.85	0.005
	Time (1 min)	0.001	0.0008	1.25	0.21
	Time (5 min)	0.0013	0.0009	1.44	0.15
	OA × Time	-0.0013	0.0005	-2.42	0.017
TEM	OA	0.0022	0.001	2.2	0.03
	Time (1 min)	0.0008	0.0007	1.14	0.26
	Time (5 min)	0.0009	0.0007	1.29	0.2
	OA × Time	-0.0005	0.0006	-0.83	0.41
SCM	OA	-0.0026	0.0011	-2.36	0.02
	Time (1 min)	-0.0003	0.0006	-0.5	0.62
	Time (5 min)	-0.0004	0.0006	-0.67	0.5
	OA × Time	0.0002	0.0004	0.5	0.62
PVM	OA	-0.0007	0.0011	-0.64	0.52
	Time (1 min)	0.0002	0.0006	0.33	0.74
	Time (5 min)	0.0001	0.0006	0.17	0.86
	OA × Time	-0.0001	0.0004	-0.25	0.8
